# Suicide among Older People in Different European Welfare Regimes: Does Economic (in)Security Have Implications for Suicide Prevention?

**DOI:** 10.3390/ijerph19127003

**Published:** 2022-06-08

**Authors:** Jing Wu, Ying Li, Margda Waern

**Affiliations:** 1 Department of Sociology and Work Science, University of Gothenburg, 40530 Gothenburg, Sweden; 2 Center for Ageing and Health, University of Gothenburg, 40530 Gothenburg, Sweden; margda.waern@neuro.gu.se; 3 School of Public Health and Community Medicine, University of Gothenburg, 40530 Gothenburg, Sweden; ying.li@gu.se; 4 Section of Psychiatry and Neurochemistry, University of Gothenburg, 40530 Gothenburg, Sweden; 5 Sahlgrenska University Hospital, 41345 Gothenburg, Sweden

**Keywords:** suicide, older people, economic insecurity, welfare regimes, suicide prevention, Europe

## Abstract

Older adult suicide rates vary widely within Europe, and differential welfare policies might contribute to this. We studied variations in economic indicators and suicide rates of people 65+ across 28 European countries and examined the effects of these indicators on suicide rates, grouping countries according to their socio-political systems and welfare regimes. Suicide data was obtained from the WHO European Mortality Database. The European Union Statistics on Income and Living Conditions and the European Union Labour Force Survey provided data on economic indicators. Linear mixed models were applied. Suicide rates ranged from 4.22/100,000 (Cyprus) to 36.37/100,000 (Hungary). Material deprivation was related to elevated suicide rates in both genders in the pooled data set and in men but not women in the Continental and Island countries. Higher ratio of median income (65+/under 65) was associated with lower likelihood of suicide in women in the South-Eastern European countries. In the Nordic region, the 65+ employment rate was associated with a decreased likelihood of suicide in men. These factors to some extent show economic insecurity against older people, which influences the likelihood of suicide. Active labor market policies and inclusive social environment may contribute to suicide prevention in this age group.

## 1. Introduction

The number of people aged 65 years and above in the European Union is projected to increase by 74.4% by the year 2060 and the 80 and above age group will grow by 163.4% [1]. The absolute number of suicides among older people is expected to increase with the ageing of the population [2]. Compared to other age groups, older people die by suicide at higher rates [3]. The suicide rate might be seen as a measure of social health of a society and welfare spending of the government [4]. Stuckler, Basu and Mckee [5] emphasized the importance of social welfare spending in maintaining the health of the population. Robust social policies to ensure adequate welfare benefits for those with low or sudden loss of income are thought to be central to offsetting the impact of the economic risk factors on suicide [6]. A potential link between economic (in)security and suicide rates has been explored in several different settings. Yip and co-authors [7] revealed that the ratio of economically inactive to active older suicide decedents is about six to one in Hong Kong. In a U.S.-based study Duberstein and colleagues [8] found an elevated risk of suicide among older people with lower incomes. In a European study Yur’vey and colleagues [9] concluded that suicide mortality is lower in countries where older people can continue to participate longer in the workforce. In a systematic review Rehkopf and Buka [10] found the association between suicide rates in different geographical areas and the socio-economic characteristics, including poverty, material deprivation, unemployment and median income. Unemployment is associated with suicide rate and the association is stronger for men than for women because job loss to some extent means the loss of social position and social ties with the world [11]. Payne and colleagues [11] explained that men as the main breadwinners may be more sensitive to deprivation and more vulnerable to distress stemming from unmet gender role; however, women without paid employment outside the home have higher suicide rates, and unpaid domestic work could be one of risks in depression and in turn leads to suicide.

Suicide rates of older people vary widely across Europe [12]; the highest figures are observed in Eastern Europe and the lowest in Southern Europe [13]. Older people are more likely to experience life events that associate with suicide, such as physical illness and functional disability [14], bereavement, widowhood [15] and social disconnectedness [16]. According to a review of the literature [17], older adults with depression are at particularly high risk; alcohol use disorders are associated with suicide in late life in some countries, but not in others, and further, previous suicidal behavior, pain and cognitive deficits have been linked to suicide in this age group.

During late life, they may experience a sharp drop in income and a depletion of savings, resulting in insufficient economic resources [18]. European social policy recognizes the older adult population as an at-risk group for social exclusion [9,19], especially social exclusion in economic resources, such as employment, income, material conditions, and pension [20]. Welfare policies exert influence on economic (in)security of older people and may contribute to the variation in suicide mortality observed across Europe. However, there is no single ‘European model’ of social protection [21], which means European countries differ considerably in both the nature and extent of their social protection systems. In turn, economic conditions among older people in Europe vary widely by country [18]. The relationship between economic factors and suicide in old age has received relatively little research attention as most studies have focused primarily on working-age populations. The aim of our study is to examine variations of suicide rates of older men and women in 28 European countries, and to analyze the effects of economic indicators on suicide rates in European regions categorized by welfare regime typologies.

## 2. Materials and Methods

### 2.1. Data Collection

Suicide statistics for men and women aged 65 and above were obtained from the World Health Organization (WHO) European Mortality Database (MDB), updated in June 2018 (https://gateway.euro.who.int/en/datasets/european-mortality-database/, accessed on 17 December 2020). The MDB is widely used for international comparisons and the assessment of the health situation and trends in European countries in an international context. In this study, economic (in)security was assessed by five indicators from the European Union Statistics on Income and Living Conditions (EU-SILC) and European Union Labor Force Survey (EU-LFS) datasets.

### 2.2. Indicators

The four indicators of economic conditions of older people and one indicator of country-level economic inequality chosen for the purpose of this study are described below:

1. Poverty risk for older persons: percentage of people aged 65+ who are at risk of poverty using 50% of the national median equivalized disposable income as the poverty threshold (EU-SILC, updated February 2018).
2. Severe material deprivation for older persons: percentage of people aged 65+ severely materially deprived (EU-SILC, updated February 2018). Severe material deprivation as one income-base measure of material well-being is defined as ‘the inability to possess the goods and services and/or engage in activities that are ordinary in the society or that are socially perceived as ‘necessities’’ [18,22].
3. Relative median income: ratio of the median equivalized disposable income of people aged 65+ to the median equivalized disposable income of those aged below 65 (EU-SILC, updated February 2018).
4. Employment rate for the age group 65+ (EU-LFS, updated April 2019).
5. Gini coefficient of equivalized disposable income (EU-SILC, updated February 2018), which measures the extent to which the distribution of income among individuals or households within a society deviates from a perfectly equal distribution. It ranges from 0 to 100, where 0 represents perfect equality (everyone has the same income) and 100 represents maximum inequality (all income is accrued by a single household) (see more info: https://ec.europa.eu/eurostat/web/products-datasets/-/tessi190, accessed on 1 June 2022).

### 2.3. Classification of Countries by Welfare Regimes

The 28 European countries studied were grouped according to the welfare regime typology introduced by Esping–Andersen [23] and developed further by different scholars [24,25,26]. Continental countries (Austria, Belgium, France, Germany, Luxembourg and the Netherlands) were characterized as conservative welfare type, Nordic countries (Denmark, Finland and Sweden) as social democratic welfare regimes, Island countries (Ireland and the UK) as the liberal type, and Southern European countries (Cyprus, Greece, Italy, Malta, Portugal and Spain) as familistic welfare models. Post-socialist European countries were subdivided into Baltic (Estonia, Latvia and Lithuania), South-Eastern European countries (Bulgaria, Croatia and Romania) and Central-Eastern European countries (Czech Republic, Hungary, Poland, Slovakia and Slovenia) based on their socio-political systems and welfare models.

### 2.4. Analytical Strategies

Our analytical strategies included two steps. First, average suicide rates (number of suicides per 100,000 for all 65+ and for men and women separately) from 2008 to 2016 were calculated for each country in order to smooth occasional fluctuations due to yearly variation of suicide rates. In a second step, as the standard statistical method to handle reported measurements (yearly information), linear mixed models were used to examine general associations between economic indicators and suicide rates for the 28 countries. In the linear mixed model, the yearly reported suicide rate was the dependent variable, and severe material deprivation, median income ratio and employment were the independent variables as fixed effect. The effect of country was modelled as a random effect because countries could be considered selected from independent samples. Then, the same linear mixed model was applied separately in each of the seven country groups to examine potential different associations between economic indicators and suicide rates in the different welfare regime typologies. The mixed model analysis was done via SAS software version 9.4 (SAS Institute, Inc., Cary, NC, USA).

## 3. Results

Seen in Table 1, the suicide rates of the population aged 65 years and older were above 36 per 100,000 in the post-socialist European countries (Hungary, 36.37 per 100,000; Lithuania, 36.12 per 100,000; Slovenia, 36.06 per 100,000), while in the Southern European group, suicide rates ranged from 4.22 per 100,000 (Cyprus) to 5.41 per 100,000 (Malta). For men in this age group, Cyprus had the lowest suicide rate (7.73 per 100,000). A tenfold higher figure was observed for men in Lithuania (74.33 per 100,000). Data for women showed the lowest suicide rate in Malta (0.77 per 100,000), and the highest rate (17.29 per 100,000) was seen in Hungary.

In Table 2, high proportions of older adults at risk of poverty were seen in Cyprus, Bulgaria and Latvia (30.74%, 30.13% and 28.63%, respectively). Material deprivation among older people (for the total population, as well as for men and women separately) was relatively more prevalent in Bulgaria, Romania and Latvia and less common in Luxembourg, Sweden and the Netherlands. The ratio of relative median income between people aged above and below 65 years was relatively lower in the Baltic countries (Estonia 0.67, Latvia 0.70 and Cyprus 0.70), and higher in Luxembourg (1.08), Hungary (1.01) and France (1.00). Relatively high employment rates were observed in persons aged 65+ in Portugal in the total population (14.10%), and in both men (20.43%) and women (9.63%). In contrast, relatively lower employment rates were seen in Slovakia (total population, 1.87%; men, 2.96% and women, 1.18%). Gini coefficients ranged from 24 to 37 among the countries studied, with the three highest figures in Lithuania (36.63), Romania (35.70) and Latvia (35.37), and the three lowest in Slovenia (24.63), Slovakia (25.20) and Czech Republic (25.07).

Results from the linear mixed models using merged data from all 28 countries showed increasing suicide rates with increasing material deprivation. This was the case for the total population 65+, as well as for both men and women (Table 3). Separate linear mixed models for each country group revealed divergent patterns with regard to indicators that showed significant impact on suicide rates. Material deprivation was related to elevated suicide rates in the Continental and Island countries in the total population and in men, but not in women. Within the South-Eastern European group, average suicide rates decreased with increasing ratio of median incomes above and below age 65 in the total group and in women, but not in men. The likelihood of suicide decreased with increasing employment rate for older men in the Nordic group.

## 4. Discussion and Conclusions

Pooled data for all the countries studied showed that material deprivation in older Europeans was associated with suicide rates in this age group. This was the case in the total population aged 65 and above, as well as in both men and women. Divorce, widowhood, and illness may occur during the process of ageing. Life changes may be accompanied by a sharp drop in income and a depletion of savings which in turn leads to material deprivation [18]. On average in the European countries, 11.2% of those aged 65+ experience material deprivation, facing problems in meeting their material needs on food, housing, transport, health and social care [18]. Significant differences in material conditions exist in Europe, both between and within countries [27]. Differences in the accessibility of social services may help to explain these disparities. Countries with more generous public pension entitlements tend to have lower rates of unmet medical care among older people [18,28]. Yur’yev et al. [29] posited that through financial aid, material resources and social service provision, high national social expenditure offers instrumental support to exceeding or straining individual’s adaptive ability and in turn reduces suicide rates.

In our study, material deprivation was positively associated with suicide rates in the population and in old men in the Islands countries. Ebbinghaus’ claims [30] might provide an explanation for this, especially as we studied suicide rates of older people since 2008 when economic crisis occurred, and therefore, the aftermath of the economic recessions could be not ignored. Ebbinghaus [30] stated that old age poverty and inequality may most likely increase as a result of reduction of welfare spending, the flexibilization of labor market and economic uncertainty. For example, although social protection provided a buffer to the economic crisis that occurred in Ireland in 2008, the financial pressures to reform public social expenditure have further amplified with the sovereign debt crisis in that country. In the Continental countries, there also existed a positive association between material deprivation and suicide rates in the population and in men in our study. In this country group, pensions in old age are directly linked to their employment status [24,31], which leads to poverty in old age among those with lower socioeconomic status on the one hand. On the other hand, in the Continental welfare model, men are supposed to work fulltime, be the primary breadwinners and provide support to the whole family [31,32]. After retirement, older men are likely to withdraw from social life and lose their social roles and as a result they have less chance to fully participate in society [29,33]. These life course transitions may lead to the significant worsening of material conditions due to inability to earn income and the need to run down accumulated assets [27], which may help to explain the elevated suicide rates of older men in this country group shown in our findings.

An inverse association between relative median income and older adult suicide was observed in the South-Eastern European countries both in the total population and in women, but not in men. Increasing life expectancy, low replacement rate from the public pension systems and little pension savings or even a lack of them may cause that an increasing number of older people are exposed to financial instability or even poverty risk in these countries, and therefore, extending working life and increasing retirement age could help to safeguard against inadequate incomes in this age group in these countries [18,34]. In our study, relative median income calculated based on the equivalized disposable income, that is, the total income of a household after tax and other deductions and that is available for spending or saving, divided by the number of household members converted into equalized adults [35]. Therefore, in the South-Eastern European countries, the relative median income ratio between women above and below 65 to some extent may indicate that older women may live with family members together in the same household. The co-residence between generations has become more frequent after the 2008 economic crisis [36,37] due to massive job losses and high unemployment rates among young and middle-aged people in Europe under the economic recessions [38]. Courtin and Avendano [36] concluded that co-residing with adult children may promote mental health in older age through frequent contact with children as well as emotional and instrumental support from/to co-residing children. Likewise, a European study also showed that due to insufficient formal childcare services offered by governments, in the South-Eastern European countries, for example, in Romania, there is a relatively high proportion of grandmothers giving daily intensive childcare than that of other countries [39]. According to Yur’yev et al. [29], intergenerational intimacy and reciprocity may help older women retain their physical and mental health and result in reduced suicide rates.

This strong inverse relationship between income and suicide rates among older women in the South-Eastern European countries observed in our study may also highlight the issue of gender differences. In general, women are disadvantaged in terms of accumulating wealth across the life course [18]. The gender pay gap is a major determinant of the higher poverty rates of women in old age compared to men and is normally translated into gender gap in pension in later life [18]. In Europe, the gender pension gap ranges from 1.8 per cent to 48.7 per cent with an average of 37.2 per cent for the people in the age group 65–79 [18,40]. Women are more likely to be employed in temporary, low-skilled and part-time jobs than men and normative expectations concerning gender roles are reinforced by public policies that place the responsibility for unpaid family work on women [18]. Flexible working hours, short-term contracts and seasonal work, and ‘zero hour’ contracts are all associated with the absence of robust occupational pension schemes and the ability to save and accumulate assets in old age, especially among older women [18,41].

We observed an inverse relationship between older adult male employment rates and suicide rates in the Nordic countries. This might be explained in part by labor market policy and age management which allow older people to participate longer in the work force. A strong correlation was previously reported between lower labor-market exit age and older adult male suicide rates in European countries [9]. In Nordic countries, return to the labor market after retirement is voluntary, based on the desire and ability of older individuals [41,42]. There is active investment in supportive workplace measures, particularly training measures for maintaining the ability of older people to work and for improving the employability of older workers. In addition, welfare spending also can eliminate age barriers in employment and integrate active employment policies into pension investments [42]. These measures enable older people to be socially included in the society and attach them with their social relations and interactions, which may result in reduced suicide rates [29,33].

Although there were no statistically significant associations between the economic factors studied and suicide rates either in the Baltic countries or in the Central-Eastern countries, the effect of these factors on the well-being of older people in these countries still cannot be ignored. In these two country groups, there is no sufficient old age pension, and many older people have to return to labor market after retirement in order to survive with low pension [18,41,43]. For the Southern European countries, according to previous research, subjective well-being factors, for example, happiness, life satisfaction and self-rated health, are more important in protecting older people from suicide compared to objective material well-being factors [31], which may explain the lack of relationship between economic factors and suicide rates in this country group in the present study.

This study had several methodological limitations that should be taken into consideration. First, data on the indicator ‘poverty-at-risk’ were not available for Croatia, Finland, Sweden and the UK. Consequently, the indicator of poverty-at-risk was not included in the linear mixed models. Second, as shown in a previous study [18], the level of material deprivation is strongly correlated with broad measures of income inequality such as the Gini coefficient. In our study, the ‘Gini coefficient’ showed strong collinearity with indicators ‘severe material deprivation’ and ‘poverty-at-risk’. As a result, the ‘Gini coefficient’ was excluded from the linear mixed models. A major limitation is that our study built on the country-level aggregate data rather than individual-level data. We lack information on social class and education which play an important role in individual chances to secure an adequate income over the life course [18] and markedly elevate the risk of material deprivation among older people [44]. Further, we have no individual-level data on other social or community factors, for example, religious affiliation, access to health care, family relationship, neighborhood, living and working conditions [9] and migration status that is also an important factor recently shown to associate with increased suicide risk in old age [45]. In addition, we lack individual-level data on clinical factors associated with suicide including depression, previous suicidal behavior, physical illness, and functional limitations, and the same applies to psychological factors such as loss of autonomy, loneliness and hopelessness [17]. Future research could include these indicators for exploring the mediating effects of individual-level factors on the relationship between economic (in)secure conditions and suicide rates among older people.

In conclusion, policy measures aimed at preventing suicide in older people in general and older men in particular in the Continental and Islands countries should take material deprivation into account. National or regional strategies to boost income in later life might contribute to a reduction in suicide rates in this age group in general and older women in particular in the South-Eastern European countries. The active labor market policies and inclusive social environment with adequate welfare benefits and pension investments may contribute to the development of programs for the prevention of suicide in this growing age group.

## Figures and Tables

**Table 1:** Suicide rates per 100,000 for people aged 65+ in 28 European countries.

Country	Suicide Rate 65+		
2008–2016	All	Male	Female
***Baltic***			
Estonia	25.13	52.33	11.43
Latvia	26.16	57.27	11.49
Lithuania	**36.12**	**74.33**	15.83
***Central-Eastern***			
Czech Republic	19.25	38.27	7.21
Hungary	**36.37**	**69.97**	**17.29**
Poland	16.76	34.27	5.58
Slovakia	14.29	31.98	3.77
Slovenia	**36.06**	**68.72**	**15.96**
***Continental***			
Austria	28.28	51.57	12.68
Belgium	23.17	37.72	13.03
France	24.27	42.96	11.64
Germany	20.11	34.53	10.19
Luxembourg	17.81	27.11	11.02
Netherlands	11.80	17.36	7.70
***Islands***			
Ireland	8.17	13.54	3.33
UK	6.12	**10.03**	2.97
***Nordic***			
Denmark	17.65	27.74	9.88
Finland	17.48	31.89	7.23
Sweden	16.40	25.92	8.74
***South-Eastern***			
Bulgaria	21.74	38.49	10.75
Croatia	32.77	60.83	**15.97**
Romania	16.19	30.41	6.43
***Southern***			
Cyprus	**4.22**	**7.73**	**1.25**
Greece	**5.27**	**9.77**	**1.68**
Italy	10.57	19.25	4.34
Malta	**5.41**	11.82	**0.77**
Portugal	21.06	39.31	8.88
Spain	12.94	22.83	5.81

**Table 2:** Economic (in)security data for older people aged 65+ in 28 European countries.

Country	At Risk of Poverty ^a^			Material Deprivation ^b^			Income Ratio ^c^			Employment ^d^			Gini ^e^
2008–2016	All	Male	Female	All	Male	Female	All	Male	Female	All	Male	Female	
***Baltic***													
Estonia	27.92	16.53	**33.59**	6.02	4.21	6.93	**0.67**	**0.72**	**0.62**	**10.99**	12.99	**10.03**	34.43
Latvia	**28.63**	**20.98**	32.30	**24.28**	**19.61**	**26.51**	**0.70**	0.76	**0.67**	8.46	11.40	7.07	**35.37**
Lithuania	20.57	12.84	24.52	20.09	16.68	21.86	0.78	0.86	0.75	5.63	8.41	4.22	**36.63**
***Central-Eastern***													
Czech Republic	6.92	**3.02**	9.79	5.08	3.61	6.14	0.82	0.83	0.80	5.02	7.24	3.47	**25.07**
Hungary	**4.97**	**3.91**	**5.56**	15.18	11.39	17.38	**1.01**	**1.07**	**0.98**	**2.02**	3.14	**1.34**	28.33
Poland	13.10	9.39	15.38	13.31	10.49	15.01	0.96	1.04	0.91	4.72	7.64	2.89	30.7
Slovakia	7.33	**4.07**	9.34	10.47	8.56	11.64	0.86	0.89	0.85	**1.87**	**2.96**	**1.18**	**25.2**
Slovenia	19.38	11.09	24.97	6.54	5.23	7.43	0.88	0.95	0.82	5.74	7.97	4.26	**24.63**
***Continental***													
Austria	15.60	12.00	18.31	2.17	1.60	2.56	0.93	0.98	0.89	5.11	7.38	3.41	27.33
Belgium	18.54	17.88	19.03	2.57	1.97	3.04	0.75	0.78	0.75	2.16	3.51	**1.13**	26.00
France	9.58	7.90	10.84	2.78	2.30	3.14	**1.00**	1.05	**0.97**	2.04	**2.84**	1.44	29.93
Germany	15.39	13.16	17.43	2.69	2.17	3.19	0.88	0.89	0.87	5.06	7.19	3.38	30.17
Luxembourg	**6.39**	5.17	**7.39**	**0.20**	**0.13**	**0.26**	**1.08**	**1.10**	**1.07**	3.26	5.16	1.95	29.20
Netherlands	**6.78**	6.46	**7.04**	**0.63**	**0.64**	**0.66**	0.87	0.89	0.86	6.49	10.33	3.30	26.60
***Islands***													
Ireland	13.69	12.72	14.50	2.68	2.26	3.02	0.85	0.88	0.83	9.42	**14.72**	4.98	30.33
UK	NA	NA	NA	1.49	1.48	1.49	0.84	0.86	0.83	9.10	12.24	6.49	31.4
***Nordic***													
Denmark	13.34	12.04	14.37	0.89	0.99	0.81	0.74	**0.76**	0.73	6.43	9.87	3.64	27.30
Finland	NA	NA	NA	1.82	1.23	2.22	0.78	0.84	0.74	4.98	7.49	3.16	25.63
Sweden	NA	NA	NA	**0.47**	**0.40**	**0.52**	0.79	0.86	0.73	7.94	11.20	5.29	25.67
***South-Eastern***													
Bulgaria	**30.13**	**22.46**	**35.38**	**50.42**	**45.82**	**53.56**	0.73	0.78	0.69	3.44	5.46	2.04	34.80
Croatia	NA	NA	NA	15.44	13.20	16.89	0.84	0.91	0.80	4.56	6.16	3.52	30.13
Romania	18.16	12.02	22.28	**29.11**	**25.69**	**31.46**	0.98	**1.09**	0.91	**11.58**	13.57	**10.27**	**35.70**
***Southern***													
Cyprus	**30.74**	**26.59**	**34.26**	7.69	6.89	8.36	**0.70**	**0.75**	**0.68**	9.71	**15.32**	4.87	33.50
Greece	17.99	16.40	19.29	14.03	11.83	15.79	0.95	0.98	0.93	3.19	5.01	1.76	33.57
Italy	16.61	13.46	18.99	8.98	7.82	9.82	0.95	0.98	0.93	3.38	6.06	1.39	32.53
Malta	19.34	19.98	18.84	5.19	4.56	5.70	0.77	0.79	0.77	3.83	7.03	1.42	27.90
Portugal	18.42	16.11	20.07	8.92	7.11	10.20	0.89	0.95	0.85	**14.10**	**20.43**	**9.63**	34.40
Spain	17.23	15.63	18.44	2.42	2.06	2.71	0.94	0.98	0.92	**1.82**	**2.49**	1.36	34.33

^a^ At risk of poverty: percentage of people aged 65+ who are at the risk of poverty using 50% of the national median equivalized disposable income as the poverty threshold (EU-SILC, updated February 2018). ^b^ Material deprivation: percentage of people aged 65+ severely materially deprived (EU-SILC, updated February 2018). ^c^ Income ratio: ratio of the median equivalized disposable income of people aged 65+ to the median equivalized disposable income of those aged below 65 (EU-SILC, updated February 2018). ^d^ Employment: employment rate for the age group 65+ (EU-LFS, updated April 2019). ^e^ Gini: Gini coefficient of equivalized disposable income (EU-SILC, updated February 2018).

**Table 3:** Linear mixed models for the impact of economic (in)security indicators on suicide rates of older people aged 65+ for 28 European countries, grouped according to welfare regime typology.

Country Groups	Variables	All			Male			Female		
		Estimate	95% CI		Estimate	95% CI		Estimate	95% CI	
**All**	Material deprivation	0.197 **	0.067	0.327	0.294 *	0.042	0.547	0.117 *	0.026	0.209
	Income ratio	−3.164	−9.875	3.548	−5.987	−18.155	6.182	−3.891	−9.350	1.569
	Employment	−0.052	−0.370	0.266	−0.089	−0.525	0.347	−0.115	−0.407	0.177
**Baltic**	Material deprivation	0.270	−0.298	0.838	0.643	−0.706	1.993	0.089	−0.188	0.366
	Income ratio	−0.256	−29.945	29.433	−8.895	−77.116	59.325	−4.501	−20.173	11.171
	Employment	0.563	−1.059	2.186	0.539	−1.792	2.870	−0.408	−1.323	0.508
**Central-Eastern**	Material deprivation	0.043	−0.345	0.431	0.108	−0.843	1.059	0.100	−0.160	0.361
	Income	−12.804	−44.296	18.688	−6.126	−69.217	56.964	−13.111	−38.420	12.198
	Employment	−0.917	−2.331	0.497	−1.107	−3.428	1.214	−1.173	−2.499	0.153
**Continental**	Material deprivation	2.036 **	0.712	3.360	3.616 ***	1.979	5.252	0.797	−0.297	1.892
	Income ratio	10.761	−11.749	33.272	7.993	−18.255	34.242	3.915	−12.808	20.637
	Employment	−0.410	−1.638	0.819	−0.711	−1.696	0.274	−0.410	−1.878	1.057
**Islands**	Material deprivation	1.680 ***	0.933	2.426	3.912 ***	2.760	5.064	−0.222	−1.252	0.807
	Income ratio	−10.356	−22.278	1.566	−1.943	−10.383	6.498	−18.110	−37.098	0.878
	Employment	0.483	−0.263	1.228	−0.422	−0.860	0.016	0.926	−0.357	2.210
**Nordic**	Material deprivation	−0.312	−1.530	0.906	0.632	−2.540	3.804	−0.034	−0.802	0.733
	Income ratio	−18.453	−37.431	0.526	1.787	−21.664	25.237	−0.782	−24.633	23.070
	Employment	−0.379	−1.017	0.258	−1.435 ***	−2.144	−0.726	−0.255	−0.937	0.427
**South-Eastern**	Material deprivation	−0.139	−0.430	0.151	−0.155	−0.593	0.283	−0.053	−0.260	0.154
	Income ratio	−29.180 *	−55.535	−2.826	−22.551	−57.648	12.547	−23.086 *	−43.133	−3.038
	Employment	0.309	−0.466	1.084	0.552	−0.557	1.660	0.143	−0.468	0.754
**Southern**	Material deprivation	0.128	−0.278	0.534	0.368	−0.652	1.388	0.024	−0.138	0.186
	Income ratio	7.723	−3.918	19.364	5.149	−18.368	28.667	3.411	−1.724	8.547
	Employment	0.174	−0.348	0.695	0.027	−0.733	0.787	0.155	−0.122	0.432

* *p* < 0.05, ** *p* < 0.01, *** *p* < 0.001.

## Data Availability

Publicly available datasets were analyzed in this study. These data can be found here: [https://gateway.euro.who.int/en/datasets/european-mortality-database/; https://ec.europa.eu/eurostat/web/microdata/european-union-statistics-on-income-and-living-conditions; https://ec.europa.eu/eurostat/web/microdata/european-union-labour-force-survey].
